# Development of Pre-Clinical Models for Evaluating the Therapeutic Potential of Candidate siRNA Targeting STAT6

**DOI:** 10.1371/journal.pone.0090338

**Published:** 2014-02-27

**Authors:** Gareth D. Healey, Jennifer A. Lockridge, Shawn Zinnen, Julian M. Hopkin, Ivan Richards, William Walker

**Affiliations:** 1 College of Medicine, Swansea University, Swansea, United Kingdom; 2 Allerna Therapeutics Ltd, Swansea University, Swansea, United Kingdom; 3 Lockridge Pharmaceutical Consulting LLC, Westminster, Colorado, United States of America; 4 Zincyte Consulting, Denver, Colorado, United States of America; 5 Sunapten Therapeutics, Kalamazoo, Michigan, United States of America; Medical Center at Seattle, United States of America

## Abstract

Developing siRNA therapeutics poses technical challenges including appropriate molecular design and testing in suitable pre-clinical models. We previously detailed sequence-selection and modification strategies for siRNA candidates targeting STAT6. Here, we describe methodology that evaluates the suitability of candidate siRNA for respiratory administration. Chemically-modified siRNA exhibited similar inhibitory activity (IC_50_) against STAT6 *in vitro* compared to unmodified siRNA and apical exposure testing with Caco-2 cell monolayers showed modification was not associated with cellular toxicity. Use of a modified RNA extraction protocol improved the sensitivity of a PCR-based bio-analytical assay (lower limit of siRNA strand quantification  =  0.01 pg/µl) which was used to demonstrate that lung distribution profiles for both siRNAs were similar following intra-tracheal administration. However, after 6 hours, modified siRNA was detected in lung tissue at concentrations >1000-fold higher than unmodified siRNA. Evaluation in a rat model of allergic inflammation confirmed the persistence of modified siRNA *in vivo*, which was detectable in broncho-alveolar lavage (BAL) fluid, BAL cells and lung tissue samples, 72 hours after dosing. Based upon the concept of respiratory allergy as a single airway disease, we considered nasal delivery as a route for respiratory targeting, evaluating an intra-nasal exposure model that involved simple dosing followed by fine dissection of the nasal cavity. Notably, endogenous STAT6 expression was invariant throughout the nasal cavities and modified siRNA persisted for at least 3 days after administration. Coupled with our previous findings showing upregulated expression of inflammatory markers in nasal samples from asthmatics, these findings support the potential of intranasal siRNA delivery. In summary, we demonstrate the successful chemical modification of STAT6 targeting siRNA, which enhanced bio-availability without cellular toxicity or reduced efficacy. We have established a robust, sensitive method for determining siRNA bio-distribution *in vivo*, and developed a nasal model to aid evaluation. Further work is warranted.

## Introduction

The mainstay of treatment for allergic asthma continues to rely on the use of anti-inflammatory corticosteroid drugs [1] that can have undesired side-effects [2], indicating the need for more selective therapeutics. In this regard, interleukin (IL)-13, acting through the signal transduction molecule - STAT6, has been implicated as a major driver of bronchial inflammation [3]–[5], prompting efforts to develop therapeutics that specifically inhibit this pathway [6]–[8]. Furthermore, consistent with the increased recognition that epithelial cells play a fundamental role in asthma pathogenesis [9], [10], it is notable that STAT6 expression in lung epithelial cells was shown to be exclusively required for IL-13-mediated pathology [11]. We therefore developed a refined therapeutic approach utilising small interfering RNA (siRNA) to specifically suppress STAT6 expression in lung epithelial cells and demonstrated that siRNA treatment can ablate the ongoing inflammatory response initiated in epithelial cells after *in vitro* exposure to IL-13 [12]. We further expanded this work by developing methodology for screening the STAT6 gene, permitting the identification of candidate therapeutic siRNA. In particular, we were able to identify cross-species active siRNA molecules that did not produce interferon responses and showed that stabilising chemical modifications could be applied to certain candidates without loss of targeting efficacy [13].

The development of new therapeutics involves significant hurdles, which with regard to asthma are particularly challenging given the limitations of current animal models [14]–[16] and the paucity of relevant biomarkers [17], [18]. Variables including pharmacokinetics and the status of target gene expression within respiratory tissue pre- and post-exposure are particularly pertinent when considering the preclinical evaluation of STAT6 siRNA. To address the latter, we recently evaluated primary human nasal epithelial cells, based on the premise that respiratory allergy is an integrated disorder of the respiratory tract [17], [19], [20], and found that such cells from asthmatic donors indeed possessed inflammatory changes consistent with disease phenotype [21]. Furthermore, biopsied nasal epithelium exhibited invariant STAT6 expression that was readily targetable with siRNA *ex vivo*, pointing to the potential utility of nasal epithelium in the pre-clinical testing of STAT6 siRNA. Challenges particularly relevant to the siRNA molecules themselves as drug candidates include accurate analysis of bio-distribution within target tissues in relation to the persistence of molecules engineered to act *in vivo* with minimal side effects [22]. Therefore, to address these issues, we describe the pre-clinical characterisation of STAT6 targeting respiratory siRNA with potential as novel therapeutics, placing particular focus on the comparative *in vivo* analysis of unmodified versus chemically-stabilised siRNA. Utilising an optimised candidate siRNA with complete homology to various mammalian species (see Methods) and a sensitive PCR-based bio-analytical assay, we compare siRNA bio-distribution within the lung of normal and allergen sensitised animals following intra-tracheal delivery and a model involving intra-nasal administration of siRNA. This approach of comparing traditional *in vivo* methodologies alongside the development of novel drug-testing platforms provides valuable, predictive pre-clinical data that should assist the optimal development of siRNA-based therapeutics intended for respiratory administration.

## Materials and Methods

### Small interfering RNA

STAT6-specific siRNA (372u) and mismatch (scrambled 372 sequence) control siRNA were synthesised using standard chemistry and annealed by Agilent Technologies, Inc. (Delaware, USA). Chemically-modified siRNA (372 m) and mismatch control (MMC) siRNA were similarly synthesised with specific modifications consisting of: deoxy-ribonucleotides substitutions, 2′O-methyl-modified adenosine/ guanine, internal 2′ fluoro-modified cytosine/ uracil and phosphorothioate linkages as detailed in Table 1. Scrambled (SC) siRNA used within the A549 lung epithelial cell experiments was Silencer Select Negative Control No. 1 siRNA (Life Technologies, Paisley, UK). Cross-species activity of 372 (u, m) siRNA is defined as complete sequence homology with human, mouse, rat, rhesus macaque and bovine STAT6 [13].

**Table 1:** Small interfering RNA sequence information.

siRNA	Strand	Sequence (5′ – 3′)
372u	Sense	GCA GGA AGA ACU CAA GUU Utt
	Anti-sense	AAA CUU GAG UUC UUC CUG Ctt
372m	Sense	^r^G^i2F^C^r^A ^r^G^r^G^r^A ^r^A^r^G^r^A ^r^A^i2F^C^i2F^U ^i2F^C^r^A^r^A ^r^G^i2F^U^i2F^U ^i2F^U*t*t
	Anti-sense	^r^A^r^A^r^A ^i2F^C^i2F^U^i2F^U ^m^G^m^A^m^G ^i2F^U^i2F^U^i2F^C ^i2F^U^i2F^U^i2F^C ^i2F^C^i2F^U^m^G ^i2F^C*t*t
MMC	Sense	^r^G^r^G^r^A ^r^G^i2F^C^r^A ^r^A^i2F^C^r^A ^i2F^U^r^G^i2F^U ^r^G^r^A^r^A ^i2F^C^r^A^i2F^U ^i2F^U*t*t
	Anti-sense	^r^A^r^A^i2F^U ^m^G^i2F^U^i2F^U ^i2F^C^m^A^i2F^C ^m^A^i2F^U^m^G ^i2F^U^i2F^U^m^G ^i2F^C^i2F^U^i2F^C ^i2F^C*t*t

Sequence information for each of the siRNA used. For unmodified siRNA (372u) all bases are RNA except for ‘tt’ overhangs, which are DNA thymidine bases. For modified siRNA (372m and MMC) the following nomenclature is used: r  =  RNA base, m = 2′ O-Methyl modification, i2F  =  internal 2′ fluoro modification, *  =  phosphorothioate linkage and bases with no prefix are DNA. MMC  =  mismatch control.

### Gene expression analysis

Real-time RT-PCR was performed using TaqMan gene expression assays: STAT6 Hs00598625_m1; GAPDH Hs99999905_m1; β-actin Hs99999903_m1 (Life Technologies). The expression of each test gene was normalised against expression of housekeeping genes, GAPDH & β-actin. Quantification was performed using a standard curve of recombinant human STAT6 and results expressed as absolute values or percentage STAT6 mRNA remaining. Percent mRNA remaining was calculated by multiplying the fold change value, derived using the method described by Pfaffl *et al* [23], by 100.

### Western blotting

Western Blotting for STAT6 and GAPDH was carried out as previously described [12]. Protein densitometry analysis was carried out using the Quantity One 1-D analysis software (Bio-Rad Laboratories Ltd, Hemel Hempstead, UK).

### Evaluation of cellular toxicity using Caco-2 cell monolayers

Cellular toxicity/permeability screening was performed as previously described [24], [25] with minor modifications. Briefly, Caco-2 cells were grown to confluence for 16 days on 1 µm filters in 24-well plates. Confluent monolayers were then exposed on their apical surface to siRNA diluted in HBSS (Sigma-Aldrich Ltd, Gillingham, UK) at the indicated concentrations (400 µl/well, in triplicate) for 6 hours at 37°C. Control monolayers consisted of cells exposed to 1% Triton x-100 (Sigma-Aldrich) in HBSS, or calcium-free phosphate buffered saline (PBS; Life Technologies) in both compartments. Following incubation, apical and basolateral chambers were sampled for siRNA quantification. To assess monolayer integrity, Lucifer Yellow was added to basolateral chambers (HBSS in apical chambers) and incubated for 30 minutes at 4°C with results reported as percent Lucifer yellow transfer. To permit RNA analysis, cells were washed, and harvested from the filters using TRI-Reagent (Sigma-Aldrich).

### Bio-analytical assay

The siRNA-specific assay was adapted and modified from published methodology [26] to enable quantification of individual siRNA strands in biological samples using separate (sense and anti-sense) stem-loop reverse transcription followed by TaqMan PCR. Plasma or lavage fluid samples were diluted (1∶50) in 1× Tris-EDTA (TE) buffer (Life Technologies) prior to stem-loop primer annealing and subsequent cDNA synthesis. To isolate siRNA from tissues, cells were lysed with TRI-Reagent (Sigma-Aldrich), and the first step (chloroform) phase separation then carried out. The aqueous phase from this separation was then diluted (1∶50) in 1× TE buffer and samples treated the same as the plasma and liquid samples. Standard curves were constructed using sense and anti-sense strands of known concentration diluted in 1× TE buffer. Stem-loop primer annealing was performed by heating the samples to 95°C for 5 min followed by stepped temperature ramping to 42°C at a rate of 10°C per 2 minutes. Stem-loop primers used for the detection of 372 (u, m) are shown in Table 1. Synthesis of cDNA was performed using the TaqMan microRNA RT synthesis kit (Life Technologies). Real-time PCR was then performed on cDNA samples in triplicate (20 µl reactions) using specific forward primers to the sense and anti-sense strands and a common reverse primer (Table 2). Detection of the PCR product was achieved using Universal library probe #77 (Roche Diagnostics, West Sussex, UK). Percent PCR efficiency was calculated using the formula: (10^−1/slope^)-1×100%.

**Table 2:** Bio-analytical assay primers.

Primer name	Primer sequence
**Stem-loop sense**	5'-GTCGTATCCAGTGCAGGGTCCGAGGTAGGCTGGTGGTGGGCACTGGATACGACAAAAAC
**Stem-loop anti-sense**	5'-GTCGTATCCAGTGCAGGGTCCGAGGTAGGCTGGTGGTGGGCACTGGATACGACAAGCAG
**372 sense strand specific forward primer**	5'–GCGCGCAGGAAGAACTCAAG (bold = locked nucleic acid chemistry)
**372 anti-sense strand specific forward primer**	5'-GCGCAAACTTGAGTTCTTCC (bold = locked nucleic acid chemistry)
**Common reverse primer**	5'-GTGCAGGGTCCGAGGT
**Universal library probe #77**	5'-GGTGGTGG

Bioanalytical assay primers used for the specific detection of the sense and anti-sense strands of 372 (unmodified and modified forms). Primers were synthesised by Eurogentec (Southampton, UK). Bold **A** & **T**  =  adenosine and thymine modified with locked nucleic acid (LNA) technology.

### Animal models

All procedures were approved by the South west Wales local research ethics committee and conducted in accordance with the Animal (Scientific Procedures) Act of 1986.

### Lung bio-distribution

Adult (10 weeks old) Brown Norway male rats (BN/RijHsd; Harlan Laboratories Inc.) rats were anesthetised with Isoflurane vapors utilising a precision vaporiser (3–5%, for approximately 5–10 min). Each rat received a single dose of siRNA in sterile saline via the intra-tracheal route (1 mg/kg). At the indicated time points blood was collected by closed cardiac puncture into a 5 ml syringe containing 100 µl of 1000 U/ml heparin (APP Pharmaceuticals, Illinois, USA). The needle was removed from the syringe and the blood added to a sterile, RNase-free polypropylene tube, gently mixed, and kept on ice until plasma was separated by centrifugation (within 30 min of blood collection). Animals were then euthanised with CO_2_ followed by decapitation before dissection to remove the lungs for further processing. Plasma was stored at –80°C and lung tissue was insufflated with, and then stored in RNA*later* (Life Technologies).

### Model of allergic inflammation

Brown Norway male rats were pre-sensitised to allergen by intraperitoneal injections of ovalbumin (10 µg) in aluminium hydroxide (10 mg) at days 0, 7 and 14. Animals, anaesthetised as described above, were treated with siRNA (2 mg/kg) in sterile saline via the intra-tracheal route on 3 consecutive days (100 µl volume, days: 18, 19 and 20 of the study). Dexamethasone (0.3 mg/kg) was used as the control reference drug. Sensitised/drug-treated animals were then exposed to aerosolised ovalbumin (10 mg/ml) using a nose-only inhalation system, for 1 hour on day 21 of the study. Plasma and broncho-alveolar lavage (BAL) were harvested 72 hours (day 24) after exposure to aerosolised ovalbumin from anaesthetised animals. Cytospin (Fisher Scientific, Loughborough, UK) preparations of cells recovered from lavage fluid were adjusted to 1×10^6^ cells/ml and stained with Speedy-Diff (Clin-Tech Ltd, Guilford, UK) for differential enumeration of eosinophils, neutrophils, monocytes and lymphocytes. Following lavage, animals were euthanised with CO_2_ followed by decapitation; lungs were removed, insufflated and fixed with 10% formalin prior to standard processing for paraffin-wax histopathology. Inflammation scoring of haematoxylin- & eosin-stained tissue sections was conducted in a randomised/ blinded fashion by assessing the presence of: goblet cell hyperplasia, peribronchial inflammation and eosinophil infiltration. Each characteristic was scored as either: 0 (not present), 1 (mild), 2 (moderate) or 3 (severe) and the mean scores for each lobe then added to give an overall characteristic score for the whole lung of each animal. The total lung inflammation score was determined by summation of the individual characteristic scores for each animal.

### Intra-nasal exposure model

Anaesthetised, as described above, adult male Brown Norway rats (BN/RijHsd) were administered 0.2, 1.0 or 2.0 mg/kg of siRNA in sterile saline using an Eppendorf pipette: total dose volume/rat/day  =  60 µl (two 15 µl doses within 1 minute to each nare). Tissue samples were collected from euthanised animals, as described above, 72 hours after the final dose by removing the skin and lower jaw from the skull and splitting at the medial suture. Separate structures collected from the exposed nasal cavity included right and left: nasal septum lining, maxillo-turbinate, naso-turbinate, ethmoid turbinates and lateral lining. For subsequent gene expression/siRNA quantification, excised nasal tissue samples were immediately placed into 1 ml of TRI-Reagent and processed accordingly.

### Statistical and data analysis

Statistical analyses were performed using IBM SPSS 19.0 (SPSS Inc, Chicago, IL, USA). Data were analysed using analysis of variance (ANOVA) with post hoc analysis performed using Bonferroni correction. Data are expressed as mean ± S.E.M and in all cases, P values <0.05 were considered significant.

## Results

### Effect of chemical modification on the in vitro efficacy of STAT6-targeting siRNA

**Figure 1:**
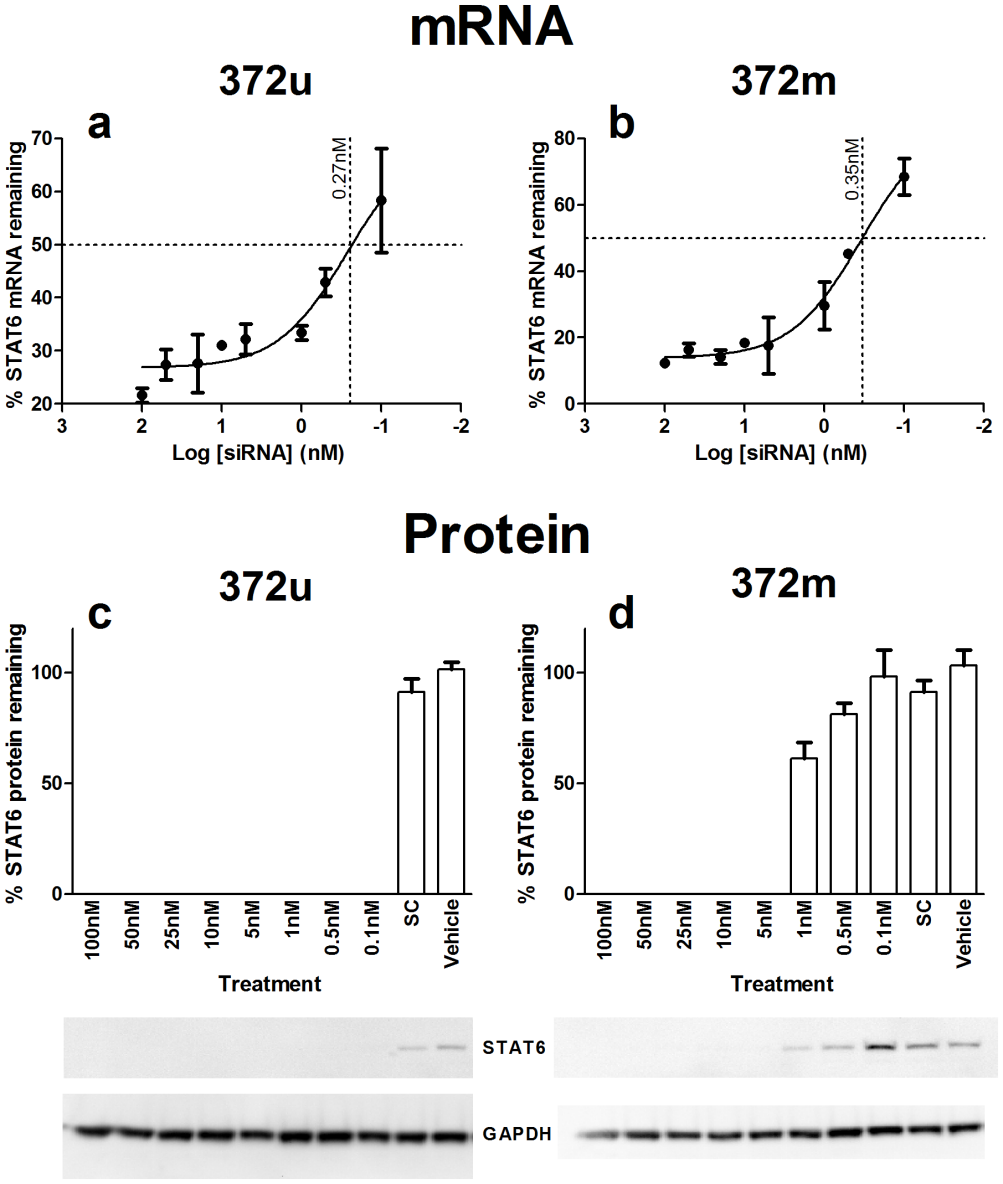
Small interfering RNA suppression of STAT6 expression *in* vitro. A549 lung epithelial cells were transfected with a range of siRNA concentrations (0.1 – 100 nM) and total RNA and protein extracted 72 hours later. The percent STAT6 mRNA remaining at each of the concentrations tested was used to calculate the 50% inhibitory concentration of 372u (a) and 372 m (b); 0.27nM and 0.35nM, respectively. Western blot analysis was then used to confirm STAT6 protein ablation, which was observed at all concentrations of 372u tested (c) and at concentrations ≥ 5 nM for 372 m (d). SC  =  Silencer Select Negative Control No. 1 siRNA, vehicle  =  transfection reagent only. Values presented are mean ± S.E.M (n  =  3).

Prior to *in vivo* testing, the specific inhibitory activity of unmodified (372u) and chemically-stabilised (372 m), cross-species (see Materials and Methods), STAT6 siRNA were verified through *in vitro* testing in human A549 lung epithelial cells as previously described [12]. Total RNA was harvested from cells 72 hours after transfection with a range of siRNA concentrations (0.1 – 100 nM) and quantitative RT-PCR used to determine the expression of STAT6 mRNA after each treatment. Data analysis showed that 372u and 372 m siRNA were comparable in their ability to suppress human STAT6 mRNA expression, exhibiting 50% inhibitory concentration (IC_50_) values of 0.27 nM and 0.35 nM respectively (Figs. 1a, b). Western blot analysis of protein extracts from siRNA-treated cells confirmed that inhibition of STAT6 mRNA correlated with a loss of protein expression. STAT6 protein expression in cells treated with 372u was undetectable at all concentrations tested (Fig. 1c), and with 372 m was undetectable at siRNA concentrations of ≥ 5.0 nM (Fig. 1d). Notably, for 372u, protein ablation was observed at concentrations below the mRNA IC_50_ value. Whilst this possibly reflects the limit of detection of the Western blotting method, it still highlights the higher efficacy of 372u compared with 372 m, as previously observed [13].

### Development and validation of a STAT6 siRNA-specific bio-analytical assay

**Figure 2:**
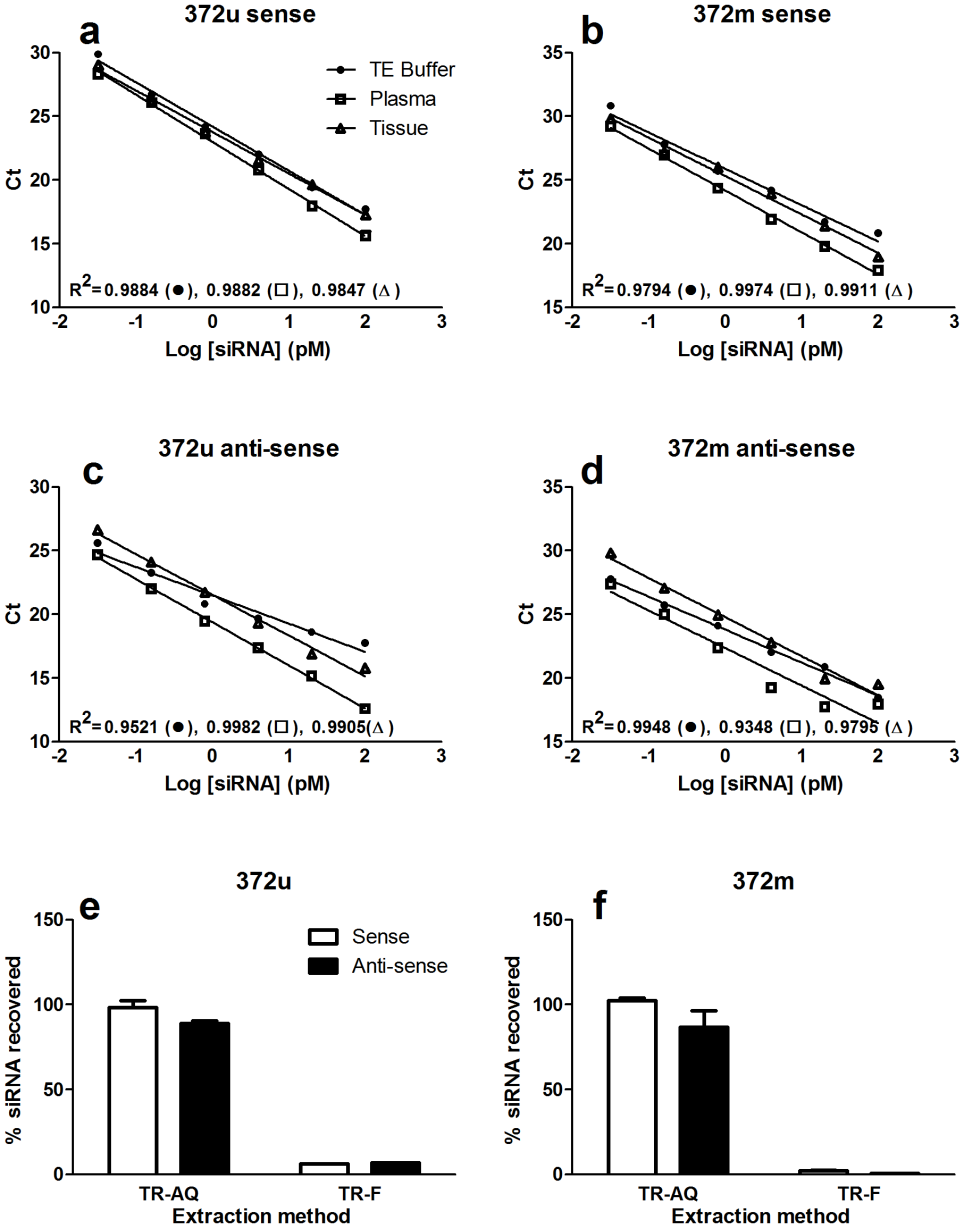
Optimisation of the bio-analytical assay. (a-d): Maintenance of bio-analytical assay sensitivity within different biological matrices. 372u or 372 m siRNA were spiked into 1 × TE buffer (•), plasma (□) or rat lung tissue extract (▵) at a range of concentrations (0.032 – 100 pg/µl) and then analysed using an RT-PCR-based bio-analytical assay. No significant difference in the threshold cycle of detection (Ct) was noted between the three matrices at any of the siRNA concentrations of tested. Shown are representative standard curves obtained for sense (a, b) and anti-sense (c, d) strand measurement. Values presented are mean (n = 3) with errors bars omitted for clarity. Percent PCR efficiency for 372u (sense) in TE buffer, plasma, or lung extract  =  94.2%, 86.3% and 102.8% respectively. Percent PCR efficiency for 372 m (sense)  =  124.2%, 101.5%, 114.8% respectively. (e, f): To compare the effect of RNA extraction methods on bio-analytical assay sensitivity, rat lung tissue was spiked with 372u (e) or 372m (f) (125 ng) and homogenised in 4M guanidine isothiocyanate (GITC) or Tri-reagent. Tri-reagent homogenised tissue was processed through the manufacturer’s recommended procedure for the extraction of RNA, either up to the chloroform phase separation step (TR-AQ) or through the entire process (TR-F). Recovery of siRNA from TR-AQ processed tissue was comparable to GITC, recovery from TR-F processed tissue was significantly lower. Values presented are mean ± S.E.M (n = 3), and represent % siRNA recovered in comparison to GITC.

In order to accurately quantify bio-distribution of siRNA we required a method that was conducive to high-throughput sample screening, capable of detecting individual siRNA strands and, given the general efficiency of siRNA-mediated gene silencing, robust enough to detect small (pico-gram) quantities in harvested tissue samples. The method used was adapted from Chen *et al* 2005 [26] and utilises separate (sense and anti-sense) stem-loop reverse transcription to detect specific siRNA strands followed by TaqMan PCR. Prior to experimental use, we performed validation testing of the assay to confirm its specificity for STAT6 siRNA and its capability to perform robustly without interference in the presence of plasma or material extracted from animal tissue. Analysis of spiked samples showed that the wide dynamic range and sensitivity (LLOQ  =  0.01 pg/µl) of the assay was not significantly affected by biological matrices such as plasma or lung tissue extract (Fig. 2a-d). Furthermore, in order to develop a simpler protocol that was compatible with simultaneous measurement of gene expression (protein and mRNA) in tissue homogenates we compared guanidine isothiocyanate (GITC) extraction with a modified TRI-Reagent extraction protocol. In this novel, truncated TRI-Reagent method, avoidance of isopropanol precipitation steps were shown to produce RNA yields equivalent to that obtained with GITC (Fig. 2e, f), we therefore employed this modified protocol in all subsequent experiments requiring siRNA and simultaneous gene expression quantification.

### Cellular toxicity analysis of 372 and 372m siRNA

**Figure 3:**
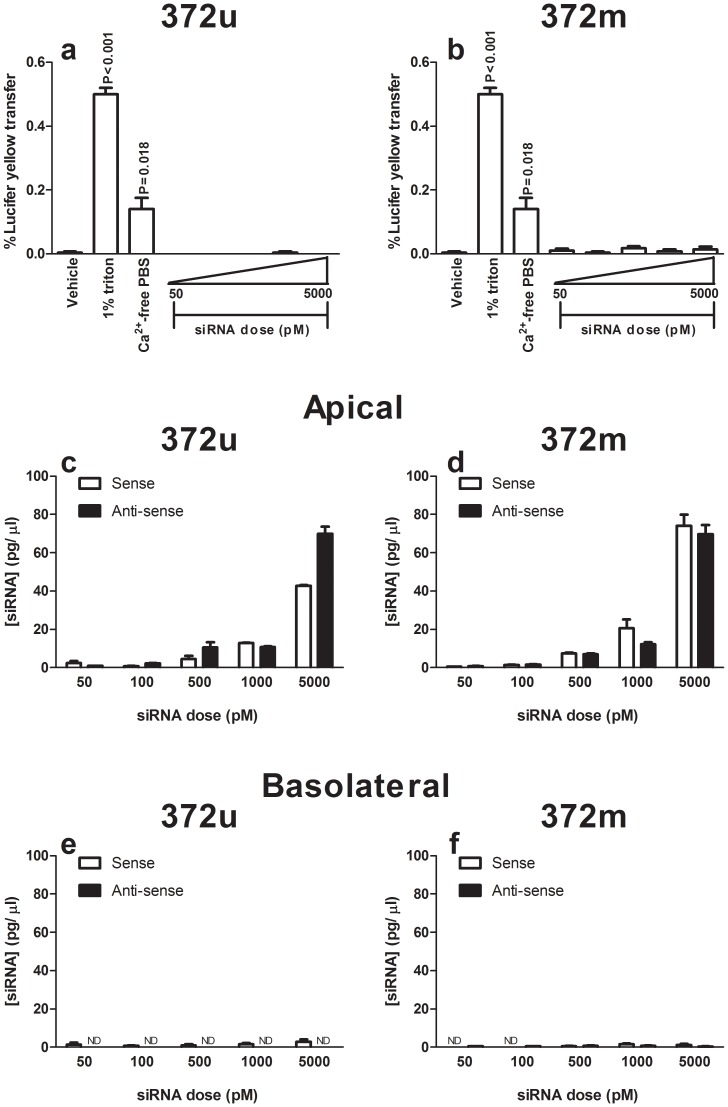
*In vitro* Caco-2 cell monolayer toxicity testing of STAT6 siRNA. Caco-2 cell monolayers were exposed to a range of siRNA concentrations (50 – 5000pM) for 6 hours and membrane integrity monitored by the transfer of Lucifer Yellow dye between the basolateral and apical compartments. No significant transfer of Lucifer Yellow dye was noted following treatment with either 372u (a) or 372m (b) compared to the vehicle control (saline). Analysis of siRNA recovered from the apical (c, d) and basolateral (e, f) compartments following treatment, showed that both 372u (c, e) and 372m (d, f) were present at ≤ 4pg/µl, LLOQ  =  0.01 pg/µl). Values presented are mean ± S.E.M (n = 3), and P-values represent comparison to the vehicle (saline) control.

Having confirmed that chemical modification did not significantly affect the suppressive activity of 372u siRNA, we wished to extend our *in vitro* analyses to include evaluation of cellular penetration and toxicity. To measure this we utilised the Caco-2 cell monolayer model which has become well established in drug permeability studies [27]. Apical exposure of cells to either unformulated siRNA (delivered in saline solution) did not disrupt the cell monolayer sufficiently to permit dye transfer between the basolateral and apical compartments, indicating that tight-junction integrity of the epithelial monolayer was maintained (Fig. 3a, b). In addition to Lucifer Yellow transfer, we utilised our PCR-based bio-analytical assay to determine the concentration of siRNA present in the apical, basolateral and cellular fractions of the Caco-2 cell assay. Consistent with the Lucifer Yellow results, bio-analytical analysis revealed that the majority of detectable siRNA was present in the apical chamber at 6 hours post-treatment (Fig. 3c, d), with very small amounts (≤ 4 pg/µl) detectable within the basolateral compartment (Fig. 3e, f) and neither siRNA being detected within the cellular compartment (data not shown). Therefore, unformulated 372u or its chemically-modified counterpart 372m, did not penetrate or have any significant detrimental effect on epithelial membrane integrity.

### Bio-distribution of STAT6 siRNA following lung exposure in normal and allergen sensitised rats

**Figure 4:**
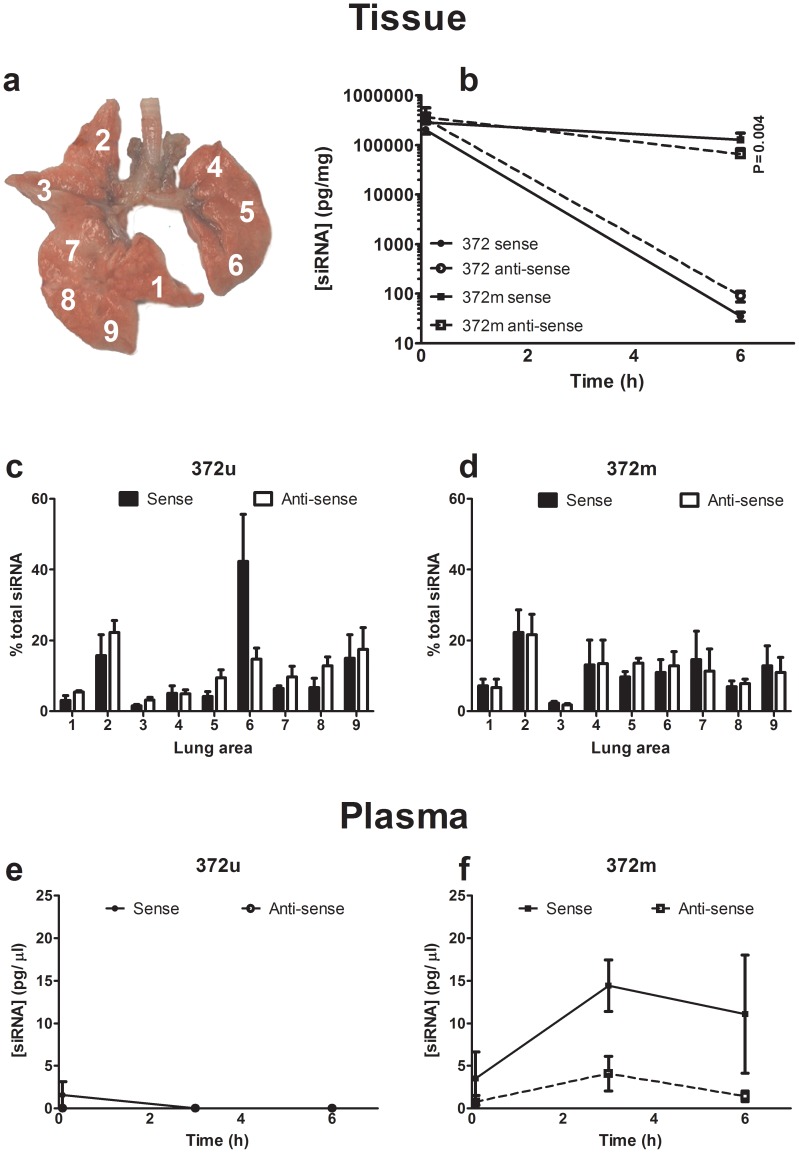
Bio-distribution and persistence of STAT6 targeting siRNA in rat lungs following intra-tracheal delivery. Normal rats were administered 372u or 372m (1 mg/kg, intra-tracheal) and siRNA quantification performed in lung tissue and plasma. Whole lung analysis showed the presence of significantly more 372m after 6 hours compared to 372u (b). 372u (c) and 372m (d) were both distributed throughout the lung 5 minutes after intra-tracheal administration. In addition, plasma concentrations of 372m (f) were significantly higher and persisted for longer than 372u (e). 1  =  right accessory lobe, 2  =  right cranial lobe, 3  =  right middle lobe, 4  =  upper left lobe, 5  =  mid left lobe, 6  =  lower left lobe, 7  =  upper right caudal lobe, 8  =  mid right caudal lobe and 9  =  lower right caudal lobe. Values presented are mean ± S.E.M (n = 3), and P-value represents comparison between 372m and 372u.

In initial bio-distribution experiments, siRNA quantification in plasma and lung tissue was performed after normal animals had received a single intra-tracheal dose of siRNA (372u or 372m, 1 mg/kg). In the absence of a suitable delivery vehicle and to avoid potential confounding factors, these experiments were performed with unformulated siRNA. Lungs were dissected into nine separate sections representing the right accessory lobe (1), right cranial lobe (2), right middle lobe (3), upper left lobe (4), mid left lobe (5), lower left lobe (6), upper right caudal lobe (7), mid right caudal lobe (8) and lower right caudal lobe (9) (Fig. 4a). Five minutes after administration both siRNA’s were distributed throughout the rat lung with notable accumulation within the right cranial lobe (2). Both sense and anti-sense strands from 372u and 372m were detectable at similar levels by bio-analytical assay (Fig. 4c, d). After 6 hours, whole lung analysis showed that 372m sense and antisense strands were detectable at a concentration >1000-fold higher than 372u (Fig. 4b; 372m = 6.5×10^4 ^pg/mg ± 3.3×10^4^, 372u = 90.1 pg/mg ± 37.5). Consistent with this observation, only 372m was detectable in plasma 6 hours post-administration (Fig. 4f).

**Figure 5:**
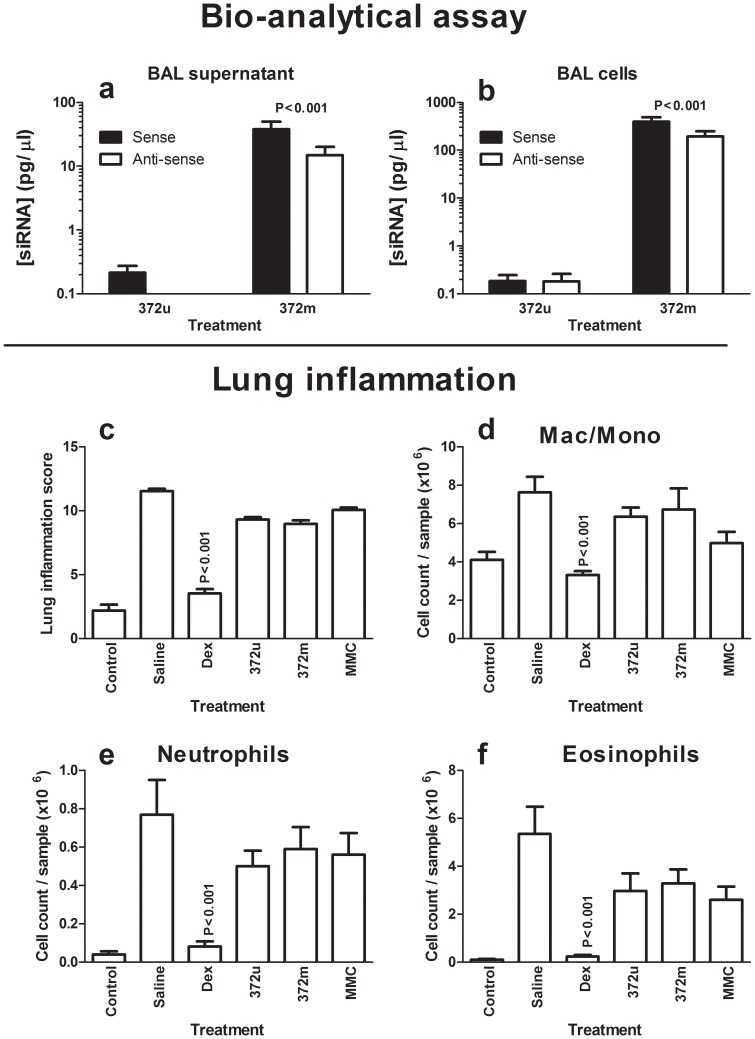
Effectiveness of STAT6 targeting siRNA within a rat model of allergic inflammation. Ovalbumin-sensitised animals were administered 372u, 372m, MMC siRNA (2 mg/kg, intra-tracheal) or dexamethasone (0.3 mg/kg) on 3 consecutive days and then aerosol challenged with ovalbumin 24 hours after the final dose. Seventy-two hours after challenge, 372m was present at significantly higher concentrations in BAL than 372u (a, b). Treatment with 372u or 372m did not reduce lung inflammation compared to the saline treated control, as evidenced by histological scoring (c) and inflammatory cell enumeration (d-f). Treatment with dexamethasone however, did significantly reduce lung inflammation following allergen challenge. Values presented are mean ± S.E.M (n = 10), and P-value represents comparison to the saline treated control.

To evaluate bio-distribution in the presence of allergic lung inflammation, siRNA were administered to ovalbumin-sensitised rats on 3 consecutive days with subsequent aerosol airway challenge with ovalbumin 24 hours after the last siRNA dose. Bio-analytical analysis of broncho-alveolar lavage (BAL) obtained from rats 72 hours after aerosolised ovalbumin challenge (6 days after first siRNA dose, 2mg/kg) showed that 372m was detectable at significantly higher levels in both BAL (Fig. 5a) and harvested cells (Fig. 5b). Histological analysis of lung sections showed that only dexamethasone treatment significantly reduced lung inflammation (goblet cell hyperplasia, peribronchial inflammation, eosinophil infiltration) following allergen challenge (Fig. 5c). Analysis of BAL cellularity corroborated this finding in that monocyte (Fig. 5d), neutrophil (Fig. 5e) and eosinophil (Fig. 5f) numbers were only significantly reduced following treatment with dexamethasone.

### Intra-nasal exposure as a pre-clinical model for assessing STAT6 siRNA

**Figure 6:**
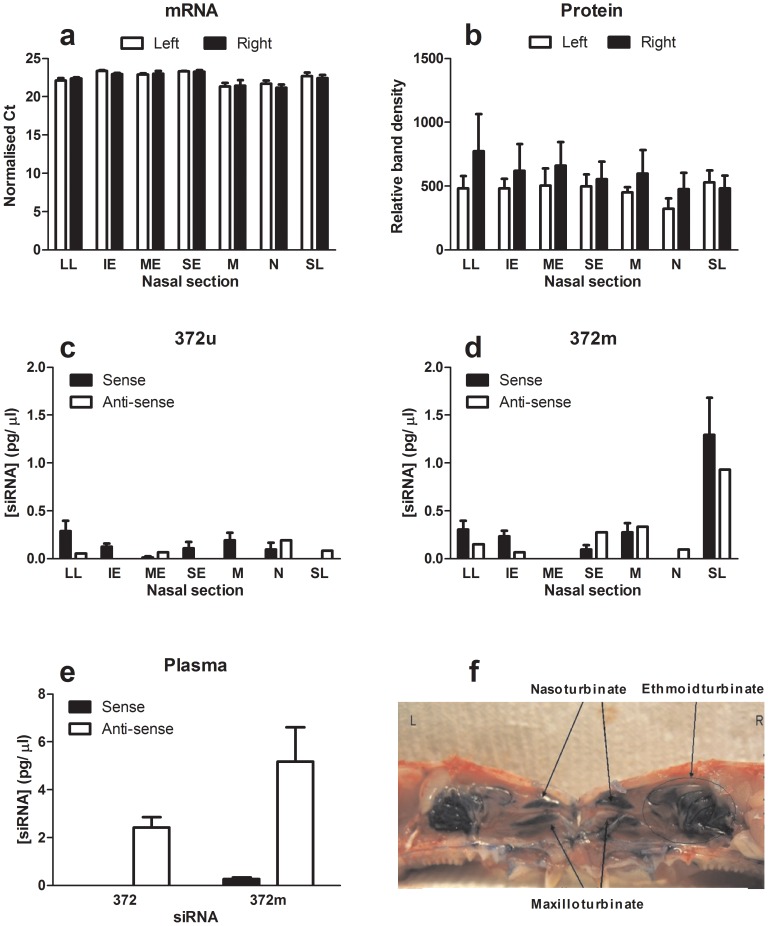
Bio-ditribution of STAT6 targeting siRNA following intranasal delivery. Fine dissection (f) followed by mRNA and protein analysis, revealed that STAT6 expression within the nasal cavity of Brown Norway rats was invariant (a, b). Anaesthetised animals were administered 2mg/kg of siRNA in sterile saline and bioanalytical analysis performed 72 hours later on fine-dissected tissue samples and plasma. Small interfering RNA was detectable throughout the nasal cavities (<0.1% original dose) with 372m present at significantly higher (P<0.001) levels in the SL compared to 372u (c, d). 372m persisted longer and was detected at significantly higher levels in rat plasma compared to 372u (e). Values presented are mean ± S.E.M (n = 6), and P-value represents comparison to 372u. Nasal sections  =  Lateral lining, LL; inferior ethmoid turbinate, IE; median ethmoid turbinate, ME; superior ethmoid turbinate, SE; maxilla-turbinate, M; naso-turbinate, N & septum lining, SL.

Given our recent findings correlating asthma with biomarker gene expression in primary human nasal epithelial cells [21], we hypothesised that intra-nasal exposure would provide a simpler, relevant approach to the analysis of STAT6 siRNA activity *in vivo*. As rat nasal cavities are predominantly lined with respiratory as well as olfactory epithelium [28], we first carried out fine dissection to determine STAT6 expression in the various nasal sections (Lateral lining, LL; inferior ethmoid turbinate, IE; median ethmoid turbinate, ME; superior ethmoid turbinate, SE; maxilla-turbinate, M; naso-turbinate, N & septum lining, SL; Fig. 6f). RT-PCR and Western blotting analysis revealed that endogenous STAT6 mRNA and STAT6 protein expression was invariant throughout the nasal cavities of untreated rats (Fig. 6a, b), consistent with our findings in both human epithelial cell lines [12] and nasal cells from human donors [21]. To allow accumulation and retention of siRNA solution within the nasal cavities, anaesthetised rats were administered small volumes of unformulated siRNA in saline using a positive displacement pipette (15 µl doses, 60 µl maximum/ animal, 2 mg/kg). Bioanalytical analysis of the nasal cavities 72 hours post-administration revealed the presence of 372u and 372m at <0.1% of the original dose (372u  =  4.3 ±2.7 ng, 372m  =  14.2 ±8.2 ng, initial dose  =  500 µg) throughout the nasal cavities (Fig. 6c, d). Of note, 372m was present at significantly (P<0.001) higher levels in the nasal septum lining (SL) compared to 372u. Consistent with this, 372m was also detectable at significantly (P<0.001) higher levels in plasma at this time point (Fig. 6e). A comparative analysis of STAT6 expression in the various nasal cavities 72 hours after administration of either 372u or 372m siRNA was also performed. When compared with saline treatment, there was a trend toward lower detectable levels of STAT6 mRNA within the inferior ethmoid turbinate of 372m-treated animals (2.0 mg/ kg) only (data not shown). However, this was not significant over three independent studies, pointing to the need for siRNA to be formulated with a suitable delivery vehicle in future efficacy studies.

## Discussion

The development of siRNA-based methods for targeting disease-associated gene expression holds great promise as the basis for new therapeutics [29], [30] and in applying this concept to allergic asthma we have demonstrated that STAT6, a key regulatory molecule within the IL-13 pathway, is amenable to targeted suppression with siRNA [12]. However, pre-clinical development of such agents face significant hurdles which include the lack of suitable methods for determining whether siRNA can target relevant tissue [22] and with regard to human asthma, the lack of appropriate animal disease models [14]–[16]. We previously detailed design strategies for identifying STAT6 siRNA candidates with cross-species homology [13]. An approach suited to the drug development process which routinely involves the use of multiple disease models within different species. We also demonstrated the utility of the nasal epithelium as a potentially viable tool for pre-clinical development, by showing candidate siRNA activity in both primary human nasal epithelium and cultures of differentiated epithelial monolayers [21]. Here, in the process of further developing our STAT6 siRNA candidates for respiratory administration, we incorporate the use of a bio-analytical method to specifically measure siRNA bio-distribution and apply this to a model involving exposure of respiratory epithelium to siRNA via intra-nasal dosing.

Accurate measurement of siRNA levels during pre-clinical testing is essential to their successful development as suitable drugs and we therefore modified the methodology of Chen *et al* 2005 [26] to develop a PCR-based bio-analytical assay with specificity to our candidate siRNA (372u, 372m). This highly sensitive assay was robust in its ability to detect either form of STAT6 siRNA within different biological matrices, including siRNA exposed tissue. To permit harvest of DNA, RNA and protein from tissue samples we incorporated TriReagent extraction into our bio-analytical assay protocol. Reliable recovery of siRNA however, required a truncated step involving harvest of RNA from the aqueous phase without isopropanol precipitation, to our knowledge this is the first report of such an approach. To-date, the majority of pre-clinical studies have relied on the use of fluorescent-/radioisotope-labelled siRNA or have utilised less sensitive hybridisation-/ELISA-based methodology to quantify siRNA levels [22]. As a pertinent comparative example, hybridisation-ELISA methodology with a lower limit of detection of 1.5 ng/ml was used to quantify siRNA in human plasma, with claims of minimal systemic bio-availability after intra-nasal exposure (150 mg/ dose) based on the non-detection of siRNA in plasma after 24 hours [31]. In contrast, the PCR-based bio-analytical assay used in this study, which was modified from previously described stem-loop RT-PCR methods for detecting small regulatory RNA [26], [32], has a detection limit of 0.01 pg/µl meaning siRNA was still detectable in rat nasal tissue and plasma 3 days after intra-nasal administration of a 500 µg dose, illustrating the greater sensitivity and utility of RT-PCR based methods for the *in vivo* quantification of siRNA.

Chemical modification of siRNA designed to inhibit degradation or off-target effects is typically associated with a concomitant reduction in silencing efficacy [33], [34]. However, we previously demonstrated that application of a chemical modification strategy favouring sense strand degradation produces siRNA which retain silencing ability without immune stimulation [13]. Here, we extend this work by showing that modified STAT6 siRNA (372m) exhibited comparable suppressive activity (IC_50_) to its unmodified counterpart (372u) and that modification was not detrimental to cells, in that neither siRNA disrupted epithelial monolayer tight-junction integrity. Furthermore, bio-distribution analysis demonstrated that both siRNAs were equally distributed throughout the lungs and that chemical modification significantly enhanced bio-availability of 372m in both lung tissue and plasma, a critical barrier to overcome in the development of siRNA therapeutics [35]–[37]. This improved bio-availability was also notable in rats with inflamed airways in that higher concentrations of 372m were recoverable from BAL. Notably, the BAL cellular fraction which consisted predominantly of inflammatory cells also contained significant amounts of 372m siRNA. Whether the detected siRNA is of actual intracellular origin or represents contaminating siRNA adhered to BAL cell surfaces, is debatable, given that unformulated siRNA exhibit poor cellular penetration characteristics [38], [39], as demonstrated by our findings showing a lack of significant siRNA transfer across Caco-2 cell monolayers. However, it is notable that macrophages, which are more receptive to siRNA uptake [40], [41], predominated in the BAL from asthmatic rats and these cells may therefore represent the source of 372m. Although unformulated 372m siRNA treatment did not attenuate allergen-induced lung inflammation in our rat model, a previous study utilising a mouse model of allergic inflammation showed that intranasal administration of unformulated STAT6 siRNA could attenuate allergic airway inflammation [42]. The reasons for this discrepancy are unclear but may reflect differences including, distinct animal models, siRNA sequences, dose and routes of administration Furthermore, previous studies using formulated siRNA to target IL-13 in a mouse model of allergic inflammation, did demonstrate efficacy (and hence cellular penetration) in that airway hyper-reactivity was ameliorated without concomitant attenuation of lung inflammation [43]. This suggests that at least in the animal models used, lung inflammation *per se* may not be a reliable indicator of targeted suppression within the IL-13 signalling pathway.

Given our recent findings of candidate siRNA activity in primary human nasal epithelium, which illustrated the potential utility of human nasal epithelium sampling in respiratory siRNA development [21]. We developed an animal model for the pre-clinical evaluation of STAT6 siRNA, that incorporated fine dissection of the nasal cavity with bio-analytical analysis to obtain a high-resolution picture of siRNA distribution. As allergic, IL-13 driven, asthma is the primary indication for therapeutic STAT6 siRNA, our rationale for this approach was based on findings that respiratory allergy is reflective of an integrated, systemic inflammatory disorder [17], [19], [20]. Furthermore, in contrast to lung delivery in which inhaled aerosol is the preferred methodology [44], intra-nasal administration provided a simpler pipette method of delivery that produced uniform siRNA distribution throughout the nasal cavity. Consistent with our previous findings in human nasal epithelial cells [21], lung epithelial, lung stromal and bronchial smooth muscle cells [12]; endogenous STAT6 expression was shown to be invariant throughout the rat nasal mucosa, suggesting that evaluation of STAT6-targeting in respiratory epithelium may be relatively uncomplicated. In these initial studies, siRNA was administered without formulation as we wished to evaluate siRNA bio-distribution in the absence of potentially confounding characteristics inherent to vehicle delivery technology. However, although bio-analytical analysis indicated possible cellular penetration, we were unable to demonstrate reproducible STAT6-targeting in nasal samples suggesting a clear requirement for a suitable delivery modality, still a major hurdle in the development of siRNA-based therapeutics [36], [38]. Indeed, models such as those described here, which enable sensitive *in vivo* measurement of siRNA distribution and target gene status, should be a useful pre-cursor to lung delivery in evaluating new delivery technologies for respiratory administration. Regarding the general relevance of intra-nasal exposure as a surrogate pre-clinical model, it is notable that nasal steroids are equally efficacious when compared with low doses of bronchial steroids for asthma therapy [45], consistent with the concept of respiratory allergy as a single airway disease [19], [46]. Given the accessibility of the nasal passages compared to the lower airways, delivering siRNA-based drugs via the nasal route may be a productive treatment strategy. Recent work by McCaskill *et al* [47] demonstrated successful target knockdown within the lungs of mice following intravenous administration. However, also notable was that approximately 70% of the dose accumulated in the liver or spleen and the majority of cells targeted within the pulmonary tissues, were endothelial cells. We would therefore argue that intravenous delivery, although attractive as a means to avoid problems associated with delivery to diseased or inflamed airways, it is an inefficient method for targeting cells of the respiratory tract and does not aid patient compliance, an important factor in the treatment of chronic respiratory disease.

In summary, we have demonstrated the successful chemical modification of STAT6 targeting siRNA, which led to significantly enhanced bio-availability without loss of efficacy or cellular toxicity. In addition, we have established a robust and highly sensitive method for determining the bio-distribution of our siRNA *in vivo*, and developed a nasal model to further aid evaluation. Further work is warranted, encompassing the optimisation of a suitable delivery modality to improve cellular uptake *in vivo* and further characterisation of cellular as well as tissue distribution.
